# 4,6-Dimeth­oxy-2-(methyl­sulfan­yl)pyrimidine

**DOI:** 10.1107/S1600536809027263

**Published:** 2009-07-18

**Authors:** Kasthuri Balasubramani, Hoong-Kun Fun

**Affiliations:** a X-ray Crystallography Unit, School of Physics, Universiti Sains Malaysia, 11800 USM, Penang, Malaysia

## Abstract

The title compound, C_7_H_10_N_2_O_2_S, is essentially planar [maximum deviation 0.018 (4) Å]. In the crystal, mol­ecules are linked into chains by C—H⋯N hydrogen bonds and the chains are arranged in layers parallel to the *ab* plane.

## Related literature

For general background to substituted pyrimidines, see: Salas *et al.* (1995 ▶); Holy *et al.* (1974 ▶); Hunt *et al.* (1980 ▶); Baker & Santi, (1965 ▶) For bond-length data, see: Allen *et al.* (1987 ▶). For the stability of the temperature controller used in the data collection, see: Cosier & Glazer (1986 ▶).
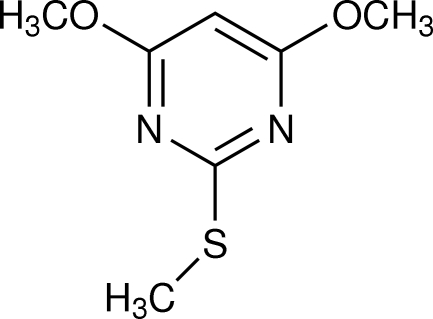


## Experimental

### 

#### Crystal data

C_7_H_10_N_2_O_2_S
*M* *_r_* = 186.23
Orthorhombic, 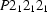

*a* = 3.9537 (2) Å
*b* = 7.1822 (4) Å
*c* = 30.5723 (15) Å
*V* = 868.14 (8) Å^3^
*Z* = 4
Mo *K*α radiation
μ = 0.33 mm^−1^
*T* = 100 K
0.55 × 0.31 × 0.05 mm

#### Data collection

Bruker SMART APEXII CCD area-detector diffractometer
Absorption correction: multi-scan (*SADABS*; Bruker, 2005 ▶) *T*_min_ = 0.838, *T*_max_ = 0.985
4467 measured reflections
1620 independent reflections
1555 reflections with *I* > 2σ(*I*)
*R*_int_ = 0.033

#### Refinement

*R*[*F* ^2^ > 2σ(*F* ^2^)] = 0.056
*wR*(*F* ^2^) = 0.133
*S* = 1.28
1620 reflections
112 parameters
H-atom parameters constrained
Δρ_max_ = 0.42 e Å^−3^
Δρ_min_ = −0.47 e Å^−3^
Absolute structure: Flack (1983 ▶), 584 Friedel pairs
Flack parameter: 0.2 (2)

### 

Data collection: *APEX2* (Bruker, 2005 ▶); cell refinement: *SAINT* (Bruker, 2005 ▶); data reduction: *SAINT*; program(s) used to solve structure: *SHELXTL* (Sheldrick, 2008 ▶); program(s) used to refine structure: *SHELXTL*; molecular graphics: *SHELXTL*; software used to prepare material for publication: *SHELXTL* and *PLATON* (Spek, 2009 ▶).

## Supplementary Material

Crystal structure: contains datablocks global, I. DOI: 10.1107/S1600536809027263/ci2850sup1.cif

Structure factors: contains datablocks I. DOI: 10.1107/S1600536809027263/ci2850Isup2.hkl

Additional supplementary materials:  crystallographic information; 3D view; checkCIF report

## Figures and Tables

**Table 1:** Hydrogen-bond geometry (Å, °)

*D*—H⋯*A*	*D*—H	H⋯*A*	*D*⋯*A*	*D*—H⋯*A*
C7—H7*A*⋯N1^i^	0.96	2.62	3.573 (6)	171

Symmetry code: (i) 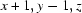.

**Fig. 1.**
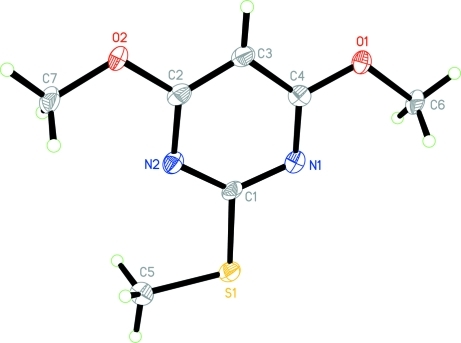
The molecular structure of the title compound, showing 50% probability displacement ellipsoids and the atom-numbering scheme.

**Fig. 2.**
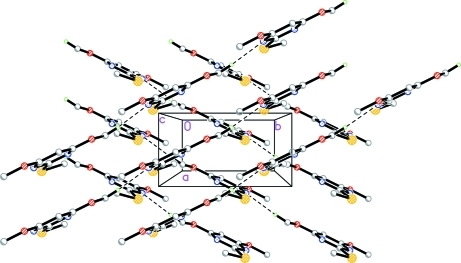
The crystal packing of the title compound, viewed along the c axis. H atoms not involved in hydrogen bonding (dashed line) have been omitted for clarity.

## supplementary crystallographic information

### Comment

Purine and pyrimidine derivatives are the constituents of nucleic acids and play important roles in many biological systems (Salas *et al.*, 1995). 2-Thiopyrimidine shows a strong bacteriostatic activity *in vitro* on *E. coli* (Holy *et al.*, 1974). Some aminopyrimidine derivatives are used as antifolate drugs (Hunt *et al.*, 1980; Baker & Santi, 1965). The crystal structure of the title compound is presented here.

The molecule (Fig.1) is essentially planar, with atom N1 deviating a maximum of 0.018 (4) Å. The bond lengths (Allen *et al.*, 1987) and angles are normal.

The molecules are linked into chains by C—H···N hydrogen bonds (Table 1). The chains are arranged in layers parallel to the *ab* plane (Fig.2).

### Experimental

Hot methanol solution (20 ml) of 4,6-dimethoxy-2-methylthiopyrimidine (46 mg, Aldrich) was warmed over a heating magnetic stirrer for 5 minutes. The resulting solution was allowed to cool slowly at room temperature. Crystals of the title compound appeared from the mother liquor after a few days.

### Refinement

H atoms were positioned geometrically [C–H = 0.93–0.96 Å] and refined using a riding model with *U*_iso_(H) = 1.2*U*_eq_(C) and 1.5*U*_eq_(methyl C). A rotating–group model was used for the methyl groups.

### Crystal data

**Table:**

C_7_H_10_N_2_O_2_S	*F*(000) = 392
*M**_r_* = 186.23	*D*_x_ = 1.425 Mg m^−^^3^
Orthorhombic, *P*2_1_2_1_2_1_	Mo *K*α radiation, λ = 0.71073 Å
Hall symbol: P 2ac 2ab	Cell parameters from 3133 reflections
*a* = 3.9537 (2) Å	θ = 2.7–30.7°
*b* = 7.1822 (4) Å	µ = 0.33 mm^−^^1^
*c* = 30.5723 (15) Å	*T* = 100 K
*V* = 868.14 (8) Å^3^	Plate, yellow
*Z* = 4	0.55 × 0.31 × 0.05 mm

### Data collection

**Table:**

Bruker SMART APEXII CCD area-detector diffractometer	1620 independent reflections
Radiation source: fine-focus sealed tube	1555 reflections with *I* > 2σ(*I*)
graphite	*R*_int_ = 0.033
φ and ω scans	θ_max_ = 26.0°, θ_min_ = 1.3°
Absorption correction: multi-scan (*SADABS*; Bruker, 2005)	*h* = −4→4
*T*_min_ = 0.838, *T*_max_ = 0.985	*k* = −6→8
4467 measured reflections	*l* = −37→37

### Refinement

**Table:**

Refinement on *F*^2^	Secondary atom site location: difference Fourier map
Least-squares matrix: full	Hydrogen site location: inferred from neighbouring sites
*R*[*F*^2^ > 2σ(*F*^2^)] = 0.056	H-atom parameters constrained
*wR*(*F*^2^) = 0.133	*w* = 1/[σ^2^(*F*_o_^2^) + 2.7239*P*] where *P* = (*F*_o_^2^ + 2*F*_c_^2^)/3
*S* = 1.28	(Δ/σ)_max_ = 0.001
1620 reflections	Δρ_max_ = 0.42 e Å^−^^3^
112 parameters	Δρ_min_ = −0.47 e Å^−^^3^
0 restraints	Absolute structure: Flack (1983), 584 Friedel pairs
Primary atom site location: structure-invariant direct methods	Flack parameter: 0.2 (2)

### Special details

**Table:**

Experimental. The crystal was placed in the cold stream of an Oxford Cyrosystems Cobra open-flow nitrogen cryostat (Cosier & Glazer, 1986) operating at 100.0 (1) K.
Geometry. All s.u.'s (except the s.u. in the dihedral angle between two l.s. planes) are estimated using the full covariance matrix. The cell s.u.'s are taken into account individually in the estimation of s.u.'s in distances, angles and torsion angles; correlations between s.u.'s in cell parameters are only used when they are defined by crystal symmetry. An approximate (isotropic) treatment of cell s.u.'s is used for estimating s.u.'s involving l.s. planes.
Refinement. Refinement of F^2^ against ALL reflections. The weighted R-factor wR and goodness of fit S are based on F^2^, conventional R-factors R are based on F, with F set to zero for negative F^2^. The threshold expression of F^2^ > σ(F^2^) is used only for calculating R-factors(gt) etc. and is not relevant to the choice of reflections for refinement. R-factors based on F^2^ are statistically about twice as large as those based on F, and R- factors based on ALL data will be even larger.

### Fractional atomic coordinates and isotropic or equivalent isotropic displacement parameters (Å^2^)

**Table:**

	*x*	*y*	*z*	*U*_iso_*/*U*_eq_	
S1	0.6163 (3)	0.16537 (16)	0.05410 (4)	0.0194 (3)	
O1	0.7269 (9)	0.2088 (4)	0.21365 (10)	0.0207 (8)	
O2	1.1384 (9)	−0.3287 (4)	0.14636 (9)	0.0200 (7)	
N1	0.6864 (9)	0.1795 (5)	0.13848 (11)	0.0140 (8)	
N2	0.8979 (11)	−0.0980 (5)	0.10377 (11)	0.0177 (8)	
C1	0.7499 (12)	0.0691 (6)	0.10392 (14)	0.0163 (9)	
C2	0.9874 (12)	−0.1607 (7)	0.14346 (14)	0.0190 (10)	
C3	0.9362 (12)	−0.0645 (6)	0.18210 (14)	0.0184 (10)	
H3A	0.9998	−0.1113	0.2092	0.022*	
C4	0.7818 (11)	0.1081 (7)	0.17698 (13)	0.0159 (9)	
C5	0.7262 (13)	−0.0160 (7)	0.01606 (14)	0.0206 (10)	
H5A	0.6651	0.0217	−0.0130	0.031*	
H5B	0.6069	−0.1279	0.0236	0.031*	
H5C	0.9653	−0.0385	0.0173	0.031*	
C6	0.5624 (13)	0.3873 (6)	0.20817 (14)	0.0200 (10)	
H6A	0.5276	0.4436	0.2363	0.030*	
H6B	0.3481	0.3699	0.1940	0.030*	
H6C	0.7023	0.4670	0.1906	0.030*	
C7	1.1937 (13)	−0.4273 (7)	0.10586 (14)	0.0210 (11)	
H7A	1.3211	−0.5385	0.1115	0.032*	
H7B	1.3170	−0.3492	0.0860	0.032*	
H7C	0.9797	−0.4598	0.0931	0.032*	

### Atomic displacement parameters (Å^2^)

**Table:**

	*U*^11^	*U*^22^	*U*^33^	*U*^12^	*U*^13^	*U*^23^
S1	0.0226 (6)	0.0161 (5)	0.0194 (5)	0.0009 (6)	−0.0013 (5)	0.0026 (5)
O1	0.0265 (19)	0.0147 (16)	0.0207 (15)	0.0071 (14)	0.0014 (14)	−0.0017 (12)
O2	0.0249 (17)	0.0127 (14)	0.0225 (14)	0.0059 (18)	0.0005 (14)	0.0001 (13)
N1	0.0059 (18)	0.0141 (17)	0.0221 (17)	−0.0036 (16)	0.0012 (13)	0.0001 (15)
N2	0.019 (2)	0.0143 (17)	0.0200 (17)	0.0012 (19)	−0.0030 (17)	0.0004 (14)
C1	0.019 (2)	0.012 (2)	0.019 (2)	−0.006 (2)	−0.0011 (19)	0.0031 (16)
C2	0.018 (2)	0.016 (2)	0.023 (2)	0.000 (2)	0.0023 (17)	0.005 (2)
C3	0.020 (3)	0.016 (2)	0.019 (2)	0.001 (2)	0.0027 (19)	0.0040 (18)
C4	0.010 (2)	0.019 (2)	0.018 (2)	−0.0014 (19)	0.0056 (17)	0.0014 (17)
C5	0.017 (3)	0.024 (2)	0.021 (2)	0.001 (2)	−0.0007 (19)	0.0000 (19)
C6	0.022 (3)	0.012 (2)	0.026 (2)	0.010 (2)	0.003 (2)	−0.0018 (18)
C7	0.021 (3)	0.017 (2)	0.026 (2)	0.007 (2)	−0.0007 (19)	−0.0013 (18)

### Geometric parameters (Å, °)

**Table:**

S1—C1	1.754 (4)	C3—C4	1.390 (7)
S1—C5	1.799 (5)	C3—H3A	0.93
O1—C4	1.352 (5)	C5—H5A	0.96
O1—C6	1.448 (5)	C5—H5B	0.96
O2—C2	1.349 (6)	C5—H5C	0.96
O2—C7	1.443 (5)	C6—H6A	0.96
N1—C4	1.338 (5)	C6—H6B	0.96
N1—C1	1.345 (6)	C6—H6C	0.96
N2—C1	1.335 (6)	C7—H7A	0.96
N2—C2	1.342 (6)	C7—H7B	0.96
C2—C3	1.384 (6)	C7—H7C	0.96
			
C1—S1—C5	101.7 (2)	S1—C5—H5B	109.5
C4—O1—C6	116.8 (3)	H5A—C5—H5B	109.5
C2—O2—C7	116.7 (3)	S1—C5—H5C	109.5
C4—N1—C1	114.4 (4)	H5A—C5—H5C	109.5
C1—N2—C2	114.5 (4)	H5B—C5—H5C	109.5
N2—C1—N1	127.9 (4)	O1—C6—H6A	109.5
N2—C1—S1	118.9 (3)	O1—C6—H6B	109.5
N1—C1—S1	113.2 (3)	H6A—C6—H6B	109.5
N2—C2—O2	118.4 (4)	O1—C6—H6C	109.5
N2—C2—C3	124.5 (4)	H6A—C6—H6C	109.5
O2—C2—C3	117.1 (4)	H6B—C6—H6C	109.5
C2—C3—C4	114.4 (4)	O2—C7—H7A	109.5
C2—C3—H3A	122.8	O2—C7—H7B	109.5
C4—C3—H3A	122.8	H7A—C7—H7B	109.5
N1—C4—O1	118.6 (4)	O2—C7—H7C	109.5
N1—C4—C3	124.4 (4)	H7A—C7—H7C	109.5
O1—C4—C3	117.0 (4)	H7B—C7—H7C	109.5
S1—C5—H5A	109.5		
			
C2—N2—C1—N1	0.9 (7)	C7—O2—C2—C3	−179.4 (4)
C2—N2—C1—S1	−179.3 (3)	N2—C2—C3—C4	−0.3 (7)
C4—N1—C1—N2	−1.1 (7)	O2—C2—C3—C4	179.6 (4)
C4—N1—C1—S1	179.1 (3)	C1—N1—C4—O1	−179.7 (4)
C5—S1—C1—N2	1.6 (4)	C1—N1—C4—C3	0.4 (6)
C5—S1—C1—N1	−178.5 (3)	C6—O1—C4—N1	0.9 (6)
C1—N2—C2—O2	179.9 (4)	C6—O1—C4—C3	−179.3 (4)
C1—N2—C2—C3	−0.2 (7)	C2—C3—C4—N1	0.1 (7)
C7—O2—C2—N2	0.4 (6)	C2—C3—C4—O1	−179.7 (4)

### Hydrogen-bond geometry (Å, °)

**Table:**

*D*—H···*A*	*D*—H	H···*A*	*D*···*A*	*D*—H···*A*
C7—H7A···N1^i^	0.96	2.62	3.573 (6)	171

Symmetry codes: (i) *x*+1, *y*−1, *z*.
